# Occupational accidents among workers within a microarea covered by the Family Health Strategy

**DOI:** 10.47626/1679-4435-2022-986

**Published:** 2024-09-24

**Authors:** Guilherme Teixeira Leite de Queiroz, Jorgana Fernanda Souza Soares, Mônica Angelim Gomes de Lima

**Affiliations:** 1 Faculdade de Medicina da Bahia, Universidade Federal da Bahia (UFBA), Salvador, BA, Brazil; 2 Department of Preventive and Social Medicine, Faculdade de Medicina da Bahia, UFBA, Salvador, BA, Brazil

**Keywords:** accidents, occupational, occupational health, primary health care, acidentes de trabalho, atenção primária à saúde, saúde do trabalhador

## Abstract

**Introduction:**

Occupational accidents are events that tend to be highly underreported in Brazil’s official reporting systems. In this context, surveys with primary data collection are important to provide a better dimensioning of these events.

**Objectives:**

To characterize workers who had a non-fatal occupational accident within the microarea of a Family Health Unit in Salvador, state of Bahia, Brazil.

**Methods:**

An observational, analytical, individual, and cross-sectional was conducted, with data collected between January and February 2020. The questionnaires were administered during home visits. The final sample consisted of 134 workers aged 18 to 65 years.

**Results:**

The incidence of occupational accidents was 6.7% (n = 9), all typical, particularly involving female workers (55.6%); aged 30 to 59 years (66.7%); with education up to high school (77.8%); self-identified as Black/Brown (100%); who received between one and two minimum monthly salaries (55.6%); single (55.6%); and with self-rated fair health (66.7%). In the population analyzed, the cumulative incidence of accidents was higher in female individuals (6.9%), with incomplete/complete high school (8.3%), self-identified as Black/Brown (7.7%), with informal employment, (7.2%), personal income of one minimum monthly salary or less (9.5%) and working > 44 hours per week (8.2%).

**Conclusions:**

A higher frequency of occupational accidents was recorded among individuals in situations of social vulnerability, which should be the focus of public policies and Occupational Health Surveillance in primary care.

## Abstract (pt)

**Introdução:**

Os acidentes de trabalho são eventos que costumam apresentar elevado sub-registro nos sistemas oficiais do país. Nesse contexto, inquéritos com coleta de dados primários são importantes para fornecer um melhor dimensionamento desses eventos.

**Objetivo:**

Caracterizar os trabalhadores vítimas de acidentes de trabalho não fatais em uma microárea adscrita a uma Unidade de Saúde da Família em Salvador, no estado da Bahia.

**Métodos:**

Foi realizado um estudo observacional, analítico, individual e transversal, e a coleta dos dados ocorreu entre janeiro e fevereiro de 2020. Os questionários foram aplicados em visitas domiciliares. A amostra final foi de 134 trabalhadores com idade entre 18 e 65 anos.

**Resultados:**

A incidência de acidentes de trabalho foi de 6,7% (n = 9), todos típicos, envolvendo, particularmente, pessoas do sexo feminino (55,6%); com idade entre 30 e 59 anos (66,7%); com escolaridade até o ensino médio (77,8%); pretas/pardas (100%); que recebiam entre um e dois salários-mínimos (55,6%); solteiras (55,6%); e com autopercepção de saúde regular (66,7%). Na população analisada, tiveram maior incidência cumulativa de acidentes os indivíduos do sexo feminino (6,9%), com ensino médio incompleto/completo (8,3%), pretos/pardos (7,7%), com vínculo de trabalho informal (7,2%), com renda de um salário-mínimo ou menos (9,5%) e carga horária semanal de mais de 44 horas (8,2%).

**Conclusões:**

Uma maior frequência de acidentes de trabalho foi registrada entre indivíduos em situação de vulnerabilidade social, que devem ser foco de políticas públicas e da Vigilância em Saúde do Trabalhador na atenção primária.

## INTRODUCTION

Occupational accidents (OAs) are acute injuries occurring during work activities that can cause a temporary or permanent reduction in the ability to perform daily activities and work tasks, and can even lead to death. OAs cover accidents that occur in the workplace (typical accidents) and those that occur on the way to or from the workplace (commuting accidents).^1,2^

More than 1.7 million OAs were recorded in Brazil between 2015 and 2017 according to the Brazilian Social Security Administration (Instituto Nacional do Seguro Social, INSS).^2^ However, these data are not very accurate in relation to the results found in population-based surveys and other studies, as they give underestimated figures.^3,4,5^ This is because the database with the largest number of OA records is the Occupational Accident Notification Information System (Sistema de Informação de Comunicação de Acidentes do Trabalho, SISCAT), which only covers formal workers employed under the Brazilian Consolidation of Labor Laws, for whom the issuance of an occupational accident report is mandatory within 24 hours for fatal cases and within 48 hours for other events. Official statistics do not include civil and military public servants, self-employed workers, employers, or informal workers.^3^

In the Notifiable Diseases Information System (Sistema de Informação de Agravos de Notificação, SINAN), reporting is mandatory only for serious accidents (fatal, involving mutilation or involving minors under 18 years of age), regardless of the employment relationship,^1^ but which are assumed to have fewer notifications than what actually occurs.^3^

OAs have major economic and social repercussions. According to the Occupational Health and Safety Observatory, based on Social Security data, between 2012 and 2020 alone, R$ 28.8 billion were spent in Brazil on sickness benefits due to OAs.^6^ Official Social Security data, however, do not take into account other losses arising from emotional and unmeasurable trauma.^7^ Furthermore, OAs burden the Brazilian Unified Health System (Sistema Único de Saúde, SUS), as they represent one of the main reasons workers receive care due to external causes,^8^ and approximately 80.7% receive some level of care in the public health system.^5^ Due to the undeniable direct and indirect social costs arising from OAs, it is necessary to invest in their investigation and prevention in the SUS Health Care Network.^7^

In 2012, the National Policy on Occupational Health (Política Nacional de Saúde do Trabalhador e da Trabalhadora, PNSTT) reinforced the importance of Primary Health Care (PHC) in surveillance and care actions for the working population.^1^ PHC plays a crucial role in occupational health care, given its characteristics as a gateway, capillarity, and organization of the SUS Health Care Network, especially at the current time of increasing unemployment and informal work.^1^

Thus, the inclusion of occupational health actions in the SUS depends directly on their incorporation into its services,^9^ since the base of these activities lies in the assigned area, the place of interaction between working users and PHC. However, a large part of the daily routine of Health Units focuses solely on care actions, without focusing on surveillance.^10^ Furthermore, there are no studies evaluating OAs within the area of a Family Health Unit (FHU) in the literature in Portuguese, with the potential of this level of care remaining underutilized in the development of occupational health actions due to a lack of knowledge of the reality of the working population.

Therefore, considering the need to produce more accurate data about OAs, especially in the primary care setting, this study aimed to characterize workers who had a non-fatal OA within the microarea of an FHU.

## METHODS

The present study is part of the macro research project entitled “Occupational Health in the Family Health Strategy in Salvador: understanding to act.”

An observational, analytical, individual, and cross-sectional, population-based study was conducted by randomly selecting a microarea within the region of the FHU - Federation, in Salvador, in the state of Bahia. All identified paid workers aged 18 to 65 years who lived in the selected microarea were included. Paid workers were defined as all those receiving monetary compensation for their occupational activity, regardless of the type of employment relationship.

The Federation is a central and populated neighborhood in the city of Salvador, with great economic heterogeneity and a population of 36,362 residents. According to the latest census, the FHU – Federation consists of five multidisciplinary health care teams that assist a population of 13,192 people divided into 29 microareas, with an average of 454.9 individuals each.

Data were collected between January and February 2020 using a questionnaire developed based on a literature review and tested in a pilot study, which was administered by trained interviewers during home visits monitored by a Community Health Worker (CHW). In cases where individuals could not complete the questionnaire at the time of the visit, another visit was scheduled at a time convenient for the participant. Households found closed more than three times or for three previously scheduled visits were considered losses. During data collection, 14 losses occurred due to households being closed after three or more visits.

The occurrence of OAs was identified by asking the following questions: “Have you had any type of accident in the past 12 months?” (no; yes). If the answer was “yes,” the participant was asked: “Were you working when you had the accident?” (no; yes). If the answer was “yes,” the OA was characterized by the following variables: type of OA (typical; commuting), presence of physical injury (no; yes), injury site (head; hand; lower limbs; other), need for medical attention (no; yes), receipt of social security benefits (no; yes), type of work performed at the time of the OA, and psychological consequences (no; yes).

The descriptor variables were sociodemographic characteristics: sex (female; male), age group (18 to 29 years; 30 to 59 years; 60 years or more), race/skin color (White; Black/Brown; other), education (incomplete/complete elementary school; incomplete/complete high school; incomplete/complete higher education); personal income (≤ 1 minimum monthly salary; > 1 and ≤ 2 minimum monthly salaries; > 2 minimum monthly salaries), and self-rated health (good, very good, or excellent; fair; bad or very bad). Data on the following occupational characteristics were also collected: type of employment relationship (categorized as formal, consisting of employees with a formal contract and statutory public servants; informal, consisting of employees without a formal contract; and self-employed), working hours (≤ 44 hours per week; > 44 hours per week), workplace (at the headquarters of a business or company; in one’s own home; in a third party’s home; outdoors; at the headquarters of one’s own business; in a public health facility), use of personal protective equipment (PPE) (no; yes), and commuter transportation (public transport; bicycle; walking; own vehicle; carpooling). The variable ‘personal income’ was categorized according to the Brazilian minimum monthly salary in 2019 (R$ 998.00). The Brazilian minimum monthly salary denotes government regulation for a minimum monthly rate paid for a worker who works, on average, 44 hours per week for four weeks in a month.

The OA incidence coefficient was calculated by dividing the number of cases by the study population multiplied by 100. For data analysis, absolute and relative frequencies were used to characterize the population and individuals who had an OA. Data were analyzed using SPSS, version 20.0. As it was a survey, inferential statistical techniques were not used.

The macro research project was approved by the Research Ethics Committee of the School of Medicine of Universidade Federal da Bahia (UFBA) (opinion no. 2,677,897 of 05/27/2018).

## RESULTS

A total of 152 workers were identified, with 14 losses due to individuals not being at home even after scheduling a visit. In addition, four (2.9%) individual questionnaires were excluded due to the lack of data on OA occurrence. Therefore, the study population consisted of 134 workers, most of whom were female (53.7%), aged 30 to 59 years (67.9%), with incomplete/complete high school, (62.7%), self-identified as Black/Brown (93.3%), with personal income between one and two minimum monthly salaries (49.6%), single (51.5%), and who self-rated their health as good, very good, or excellent (64.9%) (Table 1).

**Table 1:** Sociodemographic characteristics and self-rated health of workers participating in the study, microarea of the Family Health Unit - Federation in Salvador (state of Bahia), 2020

Variable	n	%
Sex(n =134)		
Female	72	53.7
Male	62	46.3
Age (years) (n = 134)		
18 to 29	32	23.9
30 to 59	91	679
60 or more	11	8.2
Education (n = 134)		
Incomplete/complete elementary school or less	22	16.4
Incomplete/complete high school	84	62.7
Incomplete/complete higher education	28	20.9
Race/skin color (n = 134)		
White	8	6.0
Variable	n	%
Black or Brown	125	93.3
Other	1	0.7
Personal income (minimum monthly salaries) (n = 127)		
≤ 1	42	33.1
> 1 and ≤2	63	49.6
> 2	22	17.3
Marital status (n = 134)		
Single	69	51.5
Married/consensual union	52	38.8
Separated/divorced/widowed	13	9.7
Self-rated health (n = 134)		
Good, very good, or excellent	87	64.9
Fair	43	32.1
Bad or very bad	4	3.0

Regarding occupational characteristics, most participants were informal workers (67.9%), had only one paid occupation (76.9%), worked at the headquarters of a business or company (39.8%), reported not using PPE (53.5%), commuted to work by public transport (51.5%), and worked up to 44 hours per week (63.44%) (Table 2).

**Table 2:** Occupational characteristics of workers participating in the study, microarea of the Family Health Unit - Federation in Salvador (state of Bahia), 2020

Variable	n	%
Type of employment relationship (n = 134)		
Formal	43	32.1
Informal	91	679
More than one paid occupation (n = 134)		
No	103	76.9
Yes	31	23.1
Workplace (n = 133)		
At the headquarters of a business or company	53	39.8
In one’s own home	26	19.5
In a third party’s home	25	18.8
Outdoors	19	14.3
At the headquarters of one’s own business	8	6.0
Variable	n	%
In a public health facility	2	1.5
Use of personal protective equipment (n = 101)		
Yes	47	46.5
No	54	53.5
Commuter transportation (n = 99)		
Public transport	51	51.5
Bicycle	1	1.0
Walking	36	36.4
Own vehicle	10	10.1
Carpooling	1	1.0
Weekly working hours (n = 134)		
Up to 44	85	63.4
More than 44	49	36.6

Among the workers in the study, nine OAs were identified, all typical, accounting for a cumulative incidence of 6.7%. OAs were more common among female workers (55.6%), aged 30 to 59 years (66.7%), with incomplete/complete high school (77.8%), who self-identified as Black/Brown (100%), who received between one and two minimum monthly salaries (55.6%), single (55.6%), and who self-rated their health as fair (66.7%) (Table 3). The groups that had the highest incidence of accidents in the analyzed population were female individuals (6.9%), workers with incomplete/complete high school (8.3%), and those self-identifying as Black/Brown (100%). Accidents were also more common among individuals with a personal income of one minimum monthly salary or less (9.7%), married (7.7%), and with self-rated fair health (14%).

**Table 3:** Absolute and relative frequencies and cumulative incidence according to the sociodemographic characteristics of the victims, microarea of the Family Health Unit - Federation in Salvador (state of Bahia), 2020

Variable	Occupational accident				Cumulative incidence (%)
	Yes		No		
	n	%	n	%	
Sex(n =134)					
Female	5	55.6	67	53.6	6.9
Male	4	44.4	58	46.4	6.5
Age group (years) (n = 134)					
18 to 29	3	33.3	29	23.2	9.4
30 to 59	6	66.7	85	68.0	6.6
60 or more	0	0.0	11	8.8	0.0
Education (n = 134)					
Incomplete/complete elementary school	1	11.1	21	16.8	4.5
Incomplete/complete high school	7	77.8	77	61.6	8.3
Incomplete/complete higher education	1	11.1	27	21.6	3.6
Race/skin color (n = 134)					
White	0	0.0	8	6.4	0.0
Black/Brown	9	100.0	116	92.8	7.2
Other	0	0.0	1	0.8	0.0
Personal income (minimum monthly salaries) (n = 127)					
≤ 1	4	44.4	38	32.2	9.5
>1 and≤2	5	55.6	58	49.2	79
> 2	0	0.0	22	18.6	0.0
Marital status (n = 134)			0		
Single	5	55.6	64	51.2	7.2
Married/consensual union	4	44.4	48	38.4	7.7
Separated/divorced/widowed	0	0.0	13	10.4	0.0
Self-rated health (n = 134)					
Good, very good, or excellent	3	33.3	84	67.2	3.4
Fair	6	66.7	37	29.6	14.0
Bad or very bad	0	0.0	4	3.2	0.0

Regarding occupational characteristics, OAs most frequently occurred among informal workers (77.8%), who had only one paid occupation (77.8%), worked at the headquarters of a business or company or in a third party’s home (33.3%), used PPE (55.6%), walked to work (33.3%), and worked up to 44 hours per week (55.6%) (Table 4). The groups that had the highest incidence of accidents were informal workers (7.7%), workers with only one paid occupation (4.8%), who used PPE (10.2%), and who worked more than 44 hours per week (8.2%).

**Table 4:** Absolute and relative frequencies and cumulative incidence of occupational characteristics according to the occurrence of occupational accidents, microarea of the Family Health Unit - Federation in Salvador (state of Bahia), 2020

Variable	Occupational accident				Cumulative incidence (%)
	Yes		No		
	n	%	n	%	
Type of employment relationship (n = 134)					
Formal	2	22.2	41	32.8	4.7
Informal	7	77.8	84	67.2	7.7
More than one paid occupation (n = 134)					
No	7	77.8	96	76.8	6.8
Yes	2	22.2	29	23.2	6.5
Workplace (n = 133)					
At the headquarters of a business or company	3	33.3	50	40.3	5.7
In one’s own home	2	22.2	24	19.4	77
In a third party’s home	3	33.3	22	177	12.0
Outdoors	0	0.0	19	15.3	0.0
At the headquarters of one’s own business	1	11.1	7	5.6	12.5
In a public health facility	0	0.0	2	1.6	0.0
Use of personal protective equipment (n = 101)					
Yes	5	55.6	42	45.7	10.6
No	4	44.4	50	54.3	7.4
Commuter transportation (n = 99)					
Public transport	2	33.3	49	52.7	3.9
Bicycle	1	16.7	0	0.0	100.0
Walking	3	50.0	33	35.5	8.3
Own vehicle	0	0.0	10	10.8	0.0
Carpooling	0	0.0	1	1.1	0.0
Weekly working hours (n = 134)					
Up to 44	5	55.6	80	64.0	5.9
More than 44	4	44.4	45	36.0	8.2

Workers who had an OA more frequently had physical injuries (88.9%); the hand was the main site of injury (37.5%); perforations were the main type of injury (37.5%); and approximately half of those injured required medical attention (50%). Just over one-third (37.5%) required time off work, but only 25% received any social security benefits. Only 12.5% reported having suffered family consequences after the accident, and no accident victim (0%) reported psychological problems after the accident (Table 5). Workers who had an OA also had different occupations, with two of them working in activities related to construction (bricklayer and painter’s assistant). The remaining worked as a trader/service provider, such as manicurist, practical nurse, truck unloader, receptionist, craftsperson, and upholsterer.

**Table 5:** Characterization of occupational accidents, microarea of the Family Health Unit - Federation in Salvador (state of Bahia), 2020

Variable	n	%
Physical Injury (n = 9)		
No	1	11.1
Yes	8	88.9
Injury site (n = 8)		
Head	1	12.5
Hand	3	37.5
Lower limbs	2	25
Other	2	25
Type of injury (n = 8)		
Fracture	1	12.5
Excoriation	1	12.5
Cut	2	25
Contusion	1	12.5
Perforation	3	37.5
Variable	n	%
Medical attention (n = 8)		
No	4	50
Yes	4	50
Absence from work (n = 8)		
No	5	62.5
Yes	3	37.5
Receiving sickness benefits during leave (n = 8)		
No	6	25
Yes	2	75
Family consequences after the accident (n = 8)		
No	7	12.5
Yes	1	87.5
Psychological problems after the accident (n = 8)		
No	8	0
Yes	0	100

## DISCUSSION

The incidence of OAs found in this study is close to the results from previous population-based surveys conducted in Brazil, in which the incidence ranged from 3% to 6%.^4,5^ Studies based on Social Security data have reported an incidence of approximately 1.6%.^5^ Therefore, the discrepancy between the incidence in population-based surveys and official data points to its under-enumeration, a phenomenon previously reported by other authors.^3,11,12^ The intrinsic limitation of the use of information systems that cover only formal workers employed under the Brazilian Consolidation of Labor Laws to calculate official estimates is increasingly noticeable given the evolution of the workforce profile, with the growth of informal employment.^8^

Female workers had a higher frequency of OAs in the present study. However, the male sex has been commonly associated with OAs in studies conducted in Brazil.^4^ This discrepancy between the results may be related to the fact that previous studies have been conducted with samples covering mainly formal workers, since, in some occupations, women seem to be at an equal or greater risk than men, especially in manual occupations.^12,13^ Furthermore, there is also an age-related increase in the risk of OAs among women, who are more likely to have fractures with increasing age.^13^ Another element to consider is that women report health problems more frequently than men.^12^

Regarding age, the age group 30-59 years had the highest number of accidents, while the age group 18-29 years had the highest age-specific incidence of accidents. The first finding disagrees with most of the literature, in which the frequency of these events is greater among young people,^3,4^ a condition that can be attributed to the feeling of danger invulnerability, distractions, inattention, and the lack of use of PPE in the workplace.^14^ Another factor is that young people are more commonly involved in informal work.^8^ In this study population, however, there was a high level of inclusion of workers of all ages in informal occupations, which may partly explain the finding.

Workers who reached high school, even if incomplete, and had personal income lower than two minimum monthly salaries had a higher incidence of OAs. Indirectly, education represents the type of activity performed, the occupation, and, consequently, the conditions related to occupational safety,^15^ being a factor associated with OAs in previous studies.^4,15^ One hypothesis is that a higher level of education provides greater knowledge of occupational risks and greater use of prevention strategies.^15,16^

Income can also play a similar role to that of education, being an indirect indicator of occupation, since workers employed in hazardous industries^17^ and informal workers^8^ are paid lower salaries. Thus, the finding in the present study that all workers who had an OA had personal income of up to two minimum monthly salaries is consistent with previous studies, which have also found an inversely proportional relationship between income and the occurrence of OAs.^15,18^

The present findings also support previous results regarding the relationship between race/skin color and OAs, which have shown a higher incidence of accidents among Black people.^7^ Likewise, this result is consistent with the racial composition of the city of Salvador, where Black people account for 81.8% of the population, according to 2018 data.^19^ It also corresponds to the expected result, since data were collected in a community of significant social vulnerability. In Brazil, the Black population tends to be in an unfavorable social position in the job market, with worse pay and higher unemployment rates.^19^ In the present study, conducted in a disadvantaged community in Salvador, such job market inequalities, combined with socio-spatial inequalities, may have contributed to the finding that only Black people reported having had an accident in the past 12 months.

In the present study, single people had more OAs, a result in line with that found in the city of Jequié in 2013.^8^ Married individuals generally have a strong social relationship with their partner, which can provide better socioeconomic conditions and greater social, emotional, and material support, in addition to attitudes that make them less vulnerable in all aspects of life.^20^

Workers with self-rated fair health also had more OAs. This allows us to consider again the role of precarious employment, in which workers may have worse physical and mental health conditions.^21^ Elements such as psychological stress caused by job insecurity, low wages, lack of access to health care, adverse working conditions, or even previous injuries and accidents may have an impact on self-rated health.^19^ However, cross-sectional studies, such as the present study, are unable to determine whether OAs are a consequence or cause of worse conditions and worse self-rated health in these workers, because data on exposure and outcome are collected concomitantly.

In our study population, the percentage of accidents among informal workers was almost twice that among formal workers. Informal employment is often associated with poverty, unstable jobs, asymmetry between work performed and payment received, and greater psychological and physical exposure, among other factors that may pose health risks.^12,19^ Data on accidents and health conditions of informal workers are scarce, largely due to the lack of official systems that cover this type of employment, in addition to the concentration of this type of employment in low- and middle-income countries.^12,19^

Evidence seems to point to worse health conditions among informal workers or those in precarious employment, especially in high-income countries.^19^ However, some studies indicate few differences in risk between formal and informal workers in countries such as Brazil,^3,12,19^ possibly due to poor surveillance or implementation of policies, which makes workplace conditions similar regardless of the type of employment.^12,19^

In this context, the implementation of Occupational Health Surveillance actions is crucial to combat the unhealthy conditions observed in many workplaces, leading to the promotion of health and prevention of work-related hazards, such as OAs.^22^ However, the scarcity of material and human resources, bureaucratic impediments, and management failures have posed obstacles to the effective implementation of occupational health actions,^22^ particularly workplace inspections, since most workers who had an OA worked at the company’s headquarters, demonstrating that Salvador remains in the same condition reported in 2000.^12^

From the perspective of PHC, it is worth exploring work processes, including workplaces, as an essential part of the characterization of the population and health hazards, being crucial to understand the dynamics of the coverage area and its relationships. It is also the responsibility of PHC to understand how the production and work processes function and their impact on the health of registered workers.^10^

Furthermore, the responsibilities of this level of care, as defined by the PNSTT, include the identification of the work conditions of individuals and of the economically active population as well as the mapping of productive activities and work-related hazards. Likewise, PHC must clinically manage diseases and OAs, report them, investigate the workplace, and provide labor guidance.^1^ However, in practice, these actions are neither planned nor regularly implemented in the units.^23^

Individuals who work less than 44 hours per week had a higher percentage of OAs, a result contrary to what was expected considering the relative frequencies of accidents, since overtime seems to pose a greater risk of OAs.^8^ However, other factors, such as the distribution of the working hours during the week, the shift worked (day or night), and the frequency of rest breaks, should not be disregarded and may have influenced the findings of this study.^24^ Shorter working hours, on the other hand, may be related to part-time jobs, usually associated with underemployment and precariousness.^25^

The use of PPE reduces the risk of injuries in the workplace.^26^ In the present study, however, workers using PPE had more accidents. Perhaps, in this population, workers who used PPE were under conditions of greater and, consequently, more noticeable risk. Furthermore, it is important to consider that PPE has the function of neutralizing occupational risks individually, that is, it does not completely eliminate the collective occupational risk.^27^ Therefore, in addition to PPE, collective protective equipment should preferably be implemented, as its use does not depend on individual factors.^27^

No commuting accidents were recorded, differing from previous findings in the literature, in which commuting accidents accounted for one-third of all OAs in 2013.^4^ This may be due to the frequency of home-based work or very short commuting distances, covered on foot or by public transport, in our study population. Regarding data on home-based workers, they are almost always invisible, although about 260 million people work from home worldwide.^28^ This group of workers can make almost 50% less than non-home-based workers, in addition to being less protected by regulatory legislation and, often, being subjected to excessive workloads.^28^ It is the role of PHC, through Occupational Health Surveillance of its coverage area, to map its production processes, identify this population, and intervene as necessary.^1^

Almost all accidents resulted in physical injury, with wounds (perforation, cuts, and contusions) being the most common types of injury, followed by fractures. The hands were the main affected site, a finding similar to that observed among informal traders in Jequié, who reported cuts as the main type of injury.^8^

Half of the workers with injuries caused by OAs required medical attention. This number is also similar to that of people requiring medical attention in a population-based study in Salvador, in which 49.5% of people who had an OA received first aid after the event.^29^ The present findings are also consistent with those of a study conducted in Botucatu in 2002, with a higher proportion of people who had an OA requiring medical care (80.7%).^3^

Furthermore, more than one-third of those injured were absent from work after the accident. The rate is lower than that found in Botucatu in 1997, with a rate of 69.7%,^11^ and in 2002, with 90.8% of workers requiring sick leave after an OA.^3^

No psychological or family consequences were reported after OAs. In Taiwan, a study of workers who had an OA and were hospitalized for at least 3 days in 2009 found a rate of depression or post-traumatic stress disorder of 5.2% after 12 months.^30^ However, the proportion of individuals with either of the two consequences was 0% in patients with open wounds/cuts in the upper limbs and 2.6% in those with fractures after 12 months,^30^ showing that the main injuries found in this study may imply a lower likelihood of psychological consequences.

In this study, only individuals who were employed at the time of data collection were included, which may have resulted in not recruiting people who had an OA but had lost their jobs. We chose to ask about the occurrence of OAs in the 12 months preceding the interview to avoid prevalence bias, but memory bias may have been incurred, so that less severe OAs may not have been reported by the participants. The possibility of selection bias is also undeniable for two reasons. First, because the number of workers in the microarea is unknown due to lack of control by the Family Health team. Second, because people were not always found at home, despite several attempts at different times, including weekends.

The present study allowed us to identify a general overview of the occurrence of OAs in the population and characterize their victims. Since, to date, there are no other studies of populations within a microarea, this overview allows for a more appropriate dimensioning of the problem, which can help implement future policies in the primary care setting.

## CONCLUSIONS

The occurrence of OAs in this study was analyzed based on self-reported data. The victims of the events were, most commonly, individuals aged 30 to 59 years, self-identified as Black, with informal employment, personal income of less than two minimum monthly salaries, and education up to high school. Most of the victims were single, had a self-rated fair health, and worked up to 44 hours per week at the company’s headquarters. The injured workers also had only one paid occupation, used PPE, and walked to work.

Furthermore, almost all OAs resulted in physical injury, predominantly to the hands, with wounds being the most common injuries, mainly perforation. Most of the victims required medical attention. However, only half required time off work, and only some workers received social security benefits. There was only one report of family consequences secondary to the accident, and there was no report of psychological consequences.

The data from this study support the finding that OAs are under-enumerated in official statistics. In this respect, the implementation of Occupational Health Surveillance through the incorporation of occupational health actions in the SUS and PHC, elements provided for in the 2012 PNSTT, is an essential element for tackling the problem. PHC, given its attributes, has great capacity, in conjunction with Occupational Health Surveillance, to assist in mapping the health conditions of workers and processes, as well as promoting workers’ health in their areas. Therefore, it is imperative that occupational health be prioritized on the agenda of Family Health teams, particularly in the current scenario in which workers continue to lose their rights and employment precariousness takes on new forms – and grows geometrically.
